# Rethinking Age Limits for Robotic Lung Cancer Resection: Contemporary Short-Term Outcomes in Octogenarians

**DOI:** 10.3390/cancers18193089

**Published:** 2026-09-23

**Authors:** Neelesh Bagrodia, Dhiaeddine Djabri, Tony Boualoy, Kevin Lopez, Ioana Baiu, Desmond M. D’souza, Robert E. Merritt, Peter J. Kneuertz

**Affiliations:** Division of Thoracic Surgery, Department of Surgery, The Ohio State University Wexner Medical Center, 410 W 10th Ave., Columbus, OH 43210, USAdesmond.d’souza@osumc.edu (D.M.D.);

**Keywords:** robotic thoracic surgery, octogenarians, non-small cell lung cancer

## Abstract

**Objective:** We sought to assess the outcomes of octogenarians compared to a reference group of younger patients undergoing robotic anatomic lung resection for non-small cell lung cancer (NSCLC) to better understand the postoperative expectations of this elderly patient population. **Methods:** A prospectively maintained Institutional General Thoracic Surgery Database was queried for patients aged 80 years and above (octogenarians) and 65–75 years (reference group) undergoing robotic lobectomy or segmentectomy for NSCLC between 2015 and 2026. Clinical data and postoperative outcomes were collected and compared using both unadjusted and 1:1 propensity-score matching. **Results:** In total, 822 patients were identified: 101 in the octogenarian group (mean age 82.4 years [standard deviation 2.2]) and 721 in the reference group (69.7 [3.1], *p* < 0.001). Postoperative complications that were more frequent in octogenarians included pneumothorax requiring chest tube insertion (5.9% vs. 2.2%, *p* = 0.043), atrial arrhythmias (18.8% vs. 9.2%, *p* = 0.005), and urinary tract infections (6.9% vs. 1.7%, *p* = 0.005). Air leak, atelectasis, pneumonia, respiratory failure, pleural effusion, empyema, and delirium did not differ between the groups. After propensity matching, there were no significant differences in the aforementioned postoperative outcomes. While the unadjusted length of stay was longer in octogenarians (4 days [interquartile range 3–5] vs. 3 [2–5], *p* = 0.012), this was no longer significant after matching (4 [3–5] vs. 3 [2–5], *p* = 0.369). There were no statistically significant differences in 30-day readmission rates (*p* = 0.197) or 30-day mortality (*p* = 0.781) after propensity-score matching. **Conclusions:** Among carefully selected octogenarians undergoing robotic anatomic lung resection for NSCLC, no statistically significant differences in short-term postoperative outcomes were detected compared with patients aged 65–75 years after adjustment for baseline differences. These findings support individualized surgical selection beyond chronologic age alone.

## Simple Summary

Selected octogenarians undergoing robotic anatomic lung resection for NSCLC had no statistically significant differences in postoperative complications, length of stay, 30-day readmission, or 30-day mortality compared with patients aged 65–75 years after propensity-score matching. These findings suggest that chronologic age alone may not adequately reflect short-term postoperative risk among carefully selected patients undergoing robotic lung resection.

## 1. Introduction

As life expectancy increases and the population ages, a growing proportion of patients diagnosed with non-small cell lung cancer (NSCLC) are older adults. Approximately 20% of patients diagnosed with lung cancer are now over 80 years of age, and the number of octogenarians presenting with potentially resectable disease is expected to continue to rise [1,2,3]. Among octogenarians, the potential oncologic benefit of resection must be weighed against greater physiologic vulnerability, competing non-cancer mortality, postoperative morbidity, loss of functional independence, and the potential for a prolonged recovery. As the population continues to age, determining which older patients can safely undergo curative-intent pulmonary resection has therefore become an increasingly relevant challenge in contemporary thoracic surgical practice.

The expansion of minimally invasive surgery has changed the risk-benefit profile of pulmonary resection in this population. Robotic-assisted thoracic surgery (RATS) represents the most recent evolution of minimally invasive pulmonary resection. Compared with conventional thoracotomy and video-assisted thoracoscopic surgery (VATS), robotic surgery provides enhanced three-dimensional visualization, wristed instrumentation, tremor filtration, improved ergonomics, and greater dexterity during hilar dissection and mediastinal lymphadenectomy. At present, there is a gap in evidence that specifically addresses the role of RATS in pulmonary resection for octogenarians. As robotic-assisted pulmonary resection has become the predominant minimally invasive platform at many institutions, contemporary benchmarking of robotic anatomic pulmonary resection outcomes in octogenarians is increasingly important for surgical decision-making, patient counseling, and quality improvement. Therefore, we sought to compare short-term postoperative outcomes between octogenarians and a younger reference cohort of patients aged 65 to 75 years undergoing robotic anatomic pulmonary resection for NSCLC at our institution.

## 2. Methods

### 2.1. Study Design and Patient Population

We performed a retrospective cohort study using a prospectively maintained institutional general thoracic surgery database. Adult patients undergoing robotic anatomic lung resection (lobectomy or segmentectomy) for pathologically confirmed non-small cell lung cancer (NSCLC) between February 2015 and March 2026 were identified. Patients undergoing resection for small cell carcinoma, typical or atypical carcinoid, or other neuroendocrine malignancies were excluded. Patients were stratified into an octogenarian cohort (≥80 years) and a younger reference cohort (65–75 years); patients outside these age ranges were excluded from the comparative analysis. Of 1633 robotic anatomic resections performed for NSCLC during the study period, 811 were excluded: 645 patients aged younger than 65 years and 166 patients aged 76–79 years, the latter constituting a prespecified buffer intended to maintain a clinically meaningful separation between cohorts (Figure 1). This study was deemed approved by our Institutional Review Board on 11/02/2025 (IRB#2024C0157).

### 2.2. Outcomes

The primary outcomes were 30-day mortality and length of hospital stay (days). Secondary outcomes included postoperative complications such as intensive care unit (ICU) admission, chest tube at discharge, supplemental oxygen requirement at discharge, reoperation, and 30-day readmission. Rapid discharge was defined as hospital discharge on or before postoperative day 2.

### 2.3. Statistical Analysis

Continuous variables were summarized as a mean (standard deviation) or median [interquartile range] and compared using the two-sample *t*-test or Wilcoxon rank-sum test as appropriate. Categorical variables were summarized as counts with percentages and compared using Fisher’s exact test, with a single overall *p*-value across all levels of multicategory variables (Fisher–Freeman–Halton extension).

To account for baseline differences between groups, we performed 1:1 propensity-score matching. Covariates were selected on the basis of clinical relevance to short-term postoperative outcomes, prioritizing cardiopulmonary reserve, disease stage, and operative factors. Propensity scores were estimated by multivariable logistic regression with group membership as the dependent variable and included the following covariates: sex, body mass index (BMI), hypertension, diabetes, pulmonary hypertension, interstitial lung disease, smoking status, forced expiratory volume in one second (FEV_1_) percent predicted, American Society of Anesthesiologists (ASA) physical status class, procedure (segmentectomy versus lobectomy), clinical T stage, and clinical nodal status. Missing covariate data were addressed using multiple imputation by chained equations (m = 20 imputed datasets, 10 iterations each), with predictive mean matching for continuous variables and logistic or polytomous regression for categorical variables. Within each imputed dataset, octogenarians were matched 1:1 without replacement to reference patients using nearest-neighbor matching on the propensity score. Covariate balance was assessed using absolute standardized mean differences, with a threshold of <0.1 considered indicative of adequate balance. Matched outcomes were analyzed within each imputed dataset and pooled across imputations using Rubin’s rules. Effect estimates are reported as absolute risk differences in percentage points for binary outcomes and as mean differences in days for length of stay (LOS), derived from linear models with robust standard errors fitted to the matched sample. Confidence intervals and *p*-values were obtained from the same model, ensuring that each interval and its corresponding *p*-value are mutually consistent. A two-sided *p*-value < 0.05 was considered statistically significant. All analyses were performed in R version 4.5.1 (R Foundation for Statistical Computing, Vienna, Austria).

## 3. Results

### 3.1. Patient Characteristics

Of 1633 robotic anatomic resections performed for NSCLC during the study period (Supplemental Table S1), 822 patients met this study’s inclusion criteria, including 101 octogenarians and 721 patients in the younger reference cohort (65–75 years). Compared with younger patients, octogenarians were less likely to be current smokers (8.5% vs. 31.2%; *p* < 0.001) and more likely to be never-smokers (20.2% vs. 5.2%; *p* < 0.001), had fewer cumulative smoking pack-years (median 31 vs. 40, *p* = 0.013), and they demonstrated better preoperative pulmonary function as measured by the FEV_1_ percentage predicted (median 91% vs. 84%, *p* < 0.001). Octogenarians had a greater burden of cardiac comorbidity, with higher rates of congestive heart failure (6.9% vs. 2.5%, *p* = 0.025), prior myocardial infarction (11.9% vs. 6.0%, *p* = 0.033), and a history of atrial fibrillation (9.9% vs. 4.3%, *p* = 0.025). Octogenarians also more frequently presented with clinical T2 disease (29.9% vs. 16.1%; overall clinical T stage *p* = 0.007), whereas the prevalence of clinically node-positive disease and the extent of pulmonary resection were similar between groups (Table 1).

### 3.2. Unadjusted Postoperative Outcomes

Before adjustment, most postoperative outcomes did not differ significantly between age groups, including pulmonary complications, reoperation, ICU admission, supplemental oxygen requirement at discharge, and 30-day readmission. Octogenarians experienced higher rates of postoperative atrial arrhythmia (18.8% vs. 9.2%, *p* = 0.005), urinary tract infection (6.9% vs. 1.7%, *p* = 0.005), and pneumothorax requiring chest tube (5.9% vs. 2.2%, *p* = 0.043). There were two (2%) and 43 (6%) in the octogenarian and reference groups, respectively, who required conversion to open thoracotomy during their surgery. Of the two octogenarians, both had a postoperative course complicated by atrial fibrillation, and both had an LOS of 8 days before discharge to a skilled nursing facility and home. In the primary outcome of 30-day mortality, no deaths occurred among octogenarians (0% vs. 0.3%). The median postoperative LOS was longer among octogenarians (4 [interquartile range 3–5] vs. 3 [2–5] days, *p* = 0.012), and octogenarians were less likely to achieve rapid discharge on or before postoperative day 2 (19.8% vs. 35.6%, *p* = 0.002). (Figure 2; Table 2).

### 3.3. Propensity-Matched Outcomes

Propensity-score matching yielded 101 well-balanced pairs with adequate covariate balance across all matched variables (all absolute standardized mean differences < 0.10; Supplemental Figure S1 and Supplemental Tables S2 and S3). After matching, none of the differences observed in the unadjusted analysis persisted. No difference was observed with respect to 30-day mortality (0.0% vs. 0.1%; *p* = 0.781) or in the postoperative LOS (median of 4 [3–5] vs. 3 [2–5] days; *p* = 0.369). Among secondary outcomes, no statistically significant differences were detected in major complications (8.9% vs. 8.1%; *p* = 0.871), reoperation (5.0% vs. 6.2%; *p* = 0.746), ICU admission (1.0% vs. 0.7%; *p* = 0.849), or 30-day readmission (13.9% vs. 7.7%; *p* = 0.197). The three differences observed before matching no longer reached statistical significance: atrial arrhythmia (18.8% vs. 9.8%; *p* = 0.102), urinary tract infection (6.9% vs. 1.6%; *p* = 0.077), and pneumothorax requiring chest tube (5.9% vs. 2.9%; *p* = 0.357).

Several outcomes nonetheless remained numerically higher among octogenarians, with confidence intervals compatible with clinically meaningful differences: atrial arrhythmia, urinary tract infection, delirium (6.9% vs. 2.2%; *p* = 0.168), discharge on supplemental oxygen (16.8% vs. 8.2%; *p* = 0.090), 30-day readmission, and rapid discharge (19.8% vs. 32.9%; *p* = 0.068). Because this study was not designed as an equivalence analysis and was not formally powered, the absence of statistical significance should not be interpreted as evidence of equivalent outcomes. Propensity-matched outcomes are summarized in Table 3 and Figure 3.

### 3.4. Time to Discharge

Time-to-discharge analysis demonstrated a slower rate of discharge among octogenarians in the unadjusted cohort, consistent with the observed increase in the median LOS (postoperative day 1–2 discharge 19.8% vs. 35.6%, *p* = 0.002; Figure 4A). Following propensity matching, the difference in discharge rate between age groups was no longer significant (postoperative day 1–2 discharge 19.8% vs. 32.9%, *p* = 0.068; Figure 4B).

## 4. Discussion

The present study demonstrates that robotic anatomic pulmonary resection for NSCLC can be performed safely in carefully selected octogenarians with no statistically significant differences in short-term outcomes detected relative to a younger reference population. Although octogenarians experienced higher rates of atrial arrhythmia, urinary tract infection, pneumothorax requiring chest tube reinsertion, and a modestly longer hospital length of stay on unadjusted analysis, these differences were no longer statistically significant after propensity-score matching. Likewise, no statistically significant differences were detected in pulmonary complications, reoperation, intensive care unit admission, 30-day readmission, or 30-day mortality following adjustment. Collectively, these findings suggest that chronological age alone is not an independent determinant of adverse perioperative outcomes following robotic pulmonary resection. Rather, the apparent increase in postoperative risk observed among elderly patients appears to be largely explained by differences in baseline patient characteristics and physiologic reserve.

As life expectancy continues to increase, thoracic surgeons are increasingly confronted with octogenarians presenting with potentially curable early-stage NSCLC. Contemporary evidence increasingly supports surgery as the preferred treatment for medically operable elderly patients, with multiple studies demonstrating superior long-term survival compared with radiation therapy or observation alone [2]. Minimally invasive series have reported 30-day mortality rates below 4% and 5-year survival approaching 40% to 60% among appropriately selected octogenarians [4,5,6,7,8,9,10]. Our findings build upon the growing body of literature by demonstrating that excellent short-term outcomes can also be achieved using a robotic approach in the modern era [11,12].

Perhaps the most important observation from our study is that the differences initially observed between age groups attenuated substantially and no longer reached statistical significance following adjustment for baseline characteristics. Octogenarians in our cohort had significantly better FEV_1_, fewer current smokers, and similar functional status despite a greater burden of cardiovascular comorbidity. These findings likely reflect careful surgical selection, whereby advanced age is offset by careful evaluation of physiologic reserve before offering operative intervention [13,14]. Consequently, our results reinforce the concept that physiologic rather than chronologic age should guide operative decision-making. Comprehensive assessment of pulmonary function, functional independence, frailty, and cardiovascular fitness is likely more informative than age alone when estimating perioperative risk [15,16]. Rather than serving as an exclusion criterion, chronological age should be considered one component of a multidimensional evaluation of operative candidacy [17,18].

Our findings provide contemporary benchmark outcomes demonstrating that robotic pulmonary resection can be performed with excellent short-term safety in appropriately selected octogenarians. Our parity of outcomes between octogenarians and the younger cohort may be due in part to the reduced morbidity associated with robotic surgery. While both open thoracotomy conversion patients in the octogenarian group were discharged alive from the hospital, they had a longer LOS. These results complement prior studies identifying fewer postoperative complications, reduced bleeding, shorter hospitalization, and greater rates of discharge home following RATS and VATS compared with thoracotomy [19,20,21]. Importantly, robotic procedures represented only a minority of operations during that study period, reflecting early adoption of robotic thoracic surgery. Our study extends these observations by evaluating a contemporary cohort in which robotic pulmonary resection has become the predominant minimally invasive platform at a high-volume institution.

Although octogenarians demonstrated a statistically longer median hospital stay and less frequent rapid discharge on unadjusted analysis, the absolute difference was only one day in the former, and neither was significantly different after propensity matching. Similarly, the increased rates of atrial arrhythmia, urinary tract infection, and pneumothorax observed before adjustment no longer reached statistical significance after accounting for baseline differences. These findings suggest that postoperative recovery is influenced more by patient-specific characteristics than by chronological age itself. Accordingly, efforts to further improve outcomes in elderly patients should focus less on age-based exclusion and more on optimizing modifiable perioperative risk factors, including cardiopulmonary status, perioperative rehabilitation, urinary catheter management, and prevention of postoperative atrial arrhythmias.

This study has several limitations. First, its retrospective single-center design introduces the possibility of selection bias, particularly because octogenarians selected for surgery represented a relatively fit surgical population. Consequently, our findings should not be generalized to all octogenarians with NSCLC but rather to those selected after comprehensive multidisciplinary evaluation. Second, although propensity-score matching accounted for measured baseline differences, unmeasured confounders—including frailty, sarcopenia, nutritional status, cognitive impairment, and social support—were not captured and may influence postoperative recovery. Additionally, this study was not formally powered to establish equivalence or detect differences in infrequent postoperative events. Therefore, nonsignificant findings should not be interpreted as evidence of equivalent outcomes between groups. Third, our analysis was limited to short-term perioperative outcomes and did not evaluate long-term survival, recurrence, cancer-specific outcomes, postoperative quality of life, or preservation of functional independence. Finally, as all patients underwent robotic pulmonary resection, direct comparisons with VATS or thoracotomy cannot be made. Future multicenter studies incorporating standardized geriatric assessment and long-term oncologic outcomes will further define the role of robotic pulmonary resection in this growing patient population.

## 5. Conclusions

Among carefully selected octogenarians undergoing robotic anatomic pulmonary resection for NSCLC, no statistically significant differences in short-term postoperative outcomes were detected compared to younger patients after adjustment for baseline differences. These findings add to a growing body of evidence suggesting that chronological age alone may not adequately characterize operative risk in operable NSCLC. As the population ages and robotic surgery continues to expand, individualized assessment of physiologic reserve and overall operative fitness remains important when considering surgical resection in older adults.

## Figures and Tables

**Figure 1:**
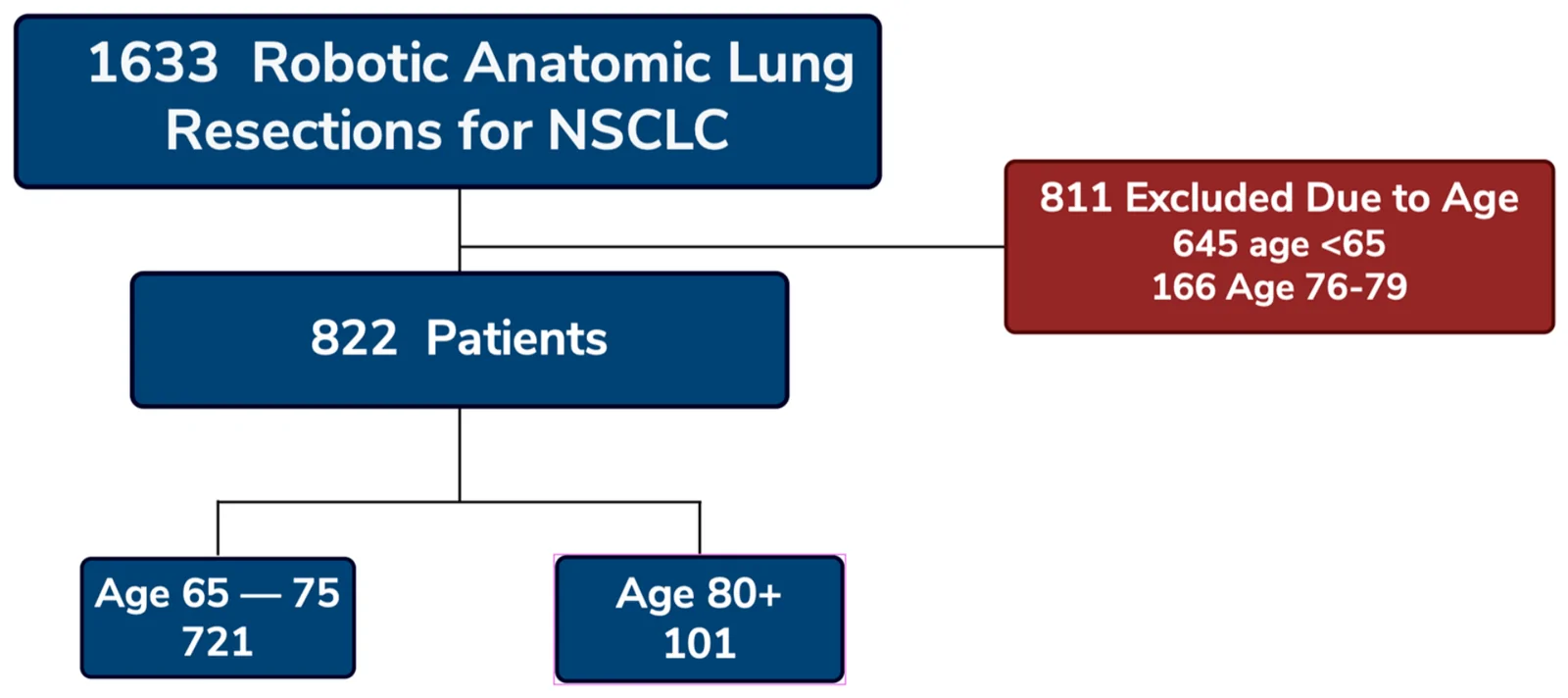
Participant flow diagram. NSCLC, non-small cell lung cancer.

**Figure 2:**
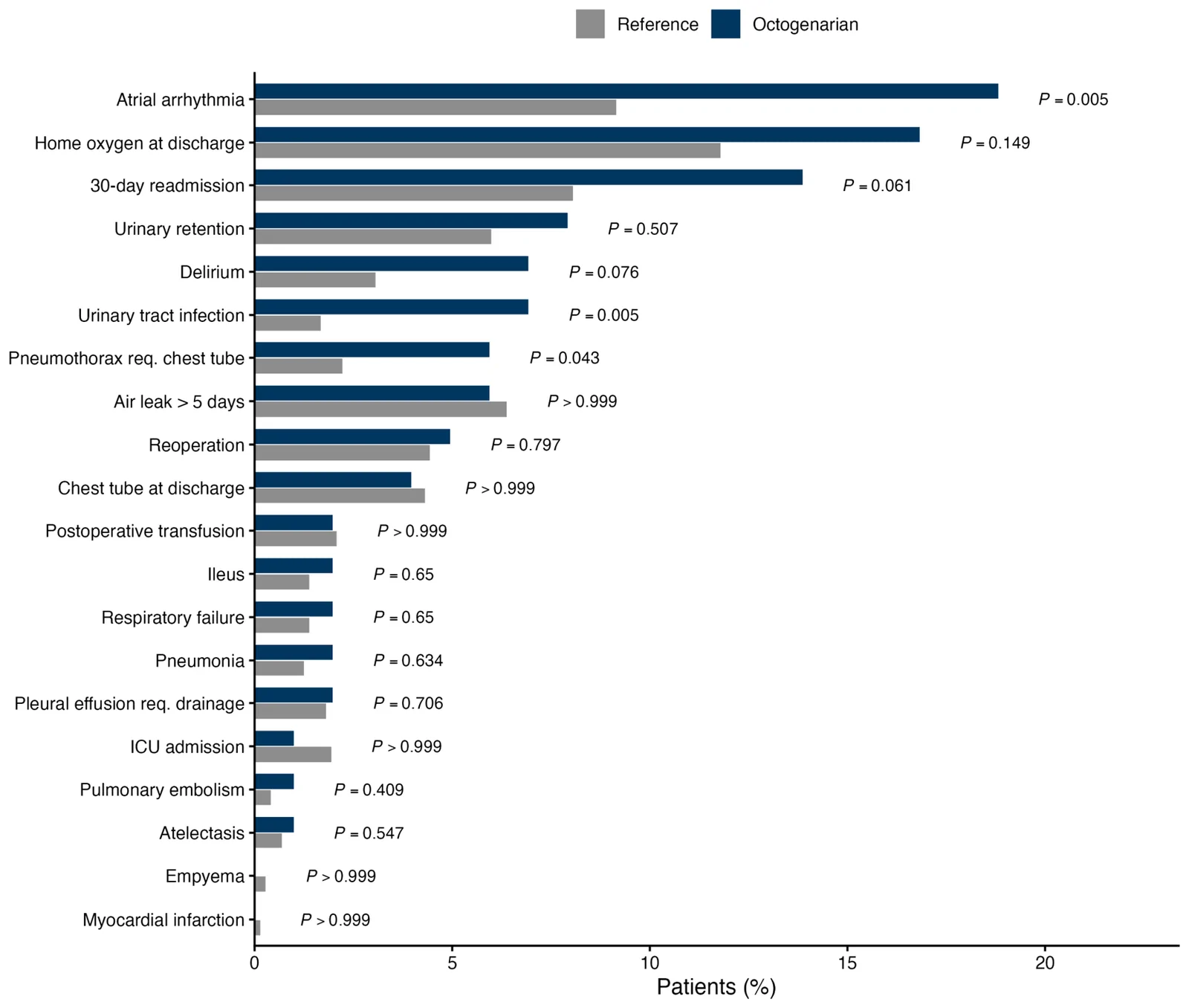
Unadjusted comparison of postoperative outcomes.

**Figure 3:**
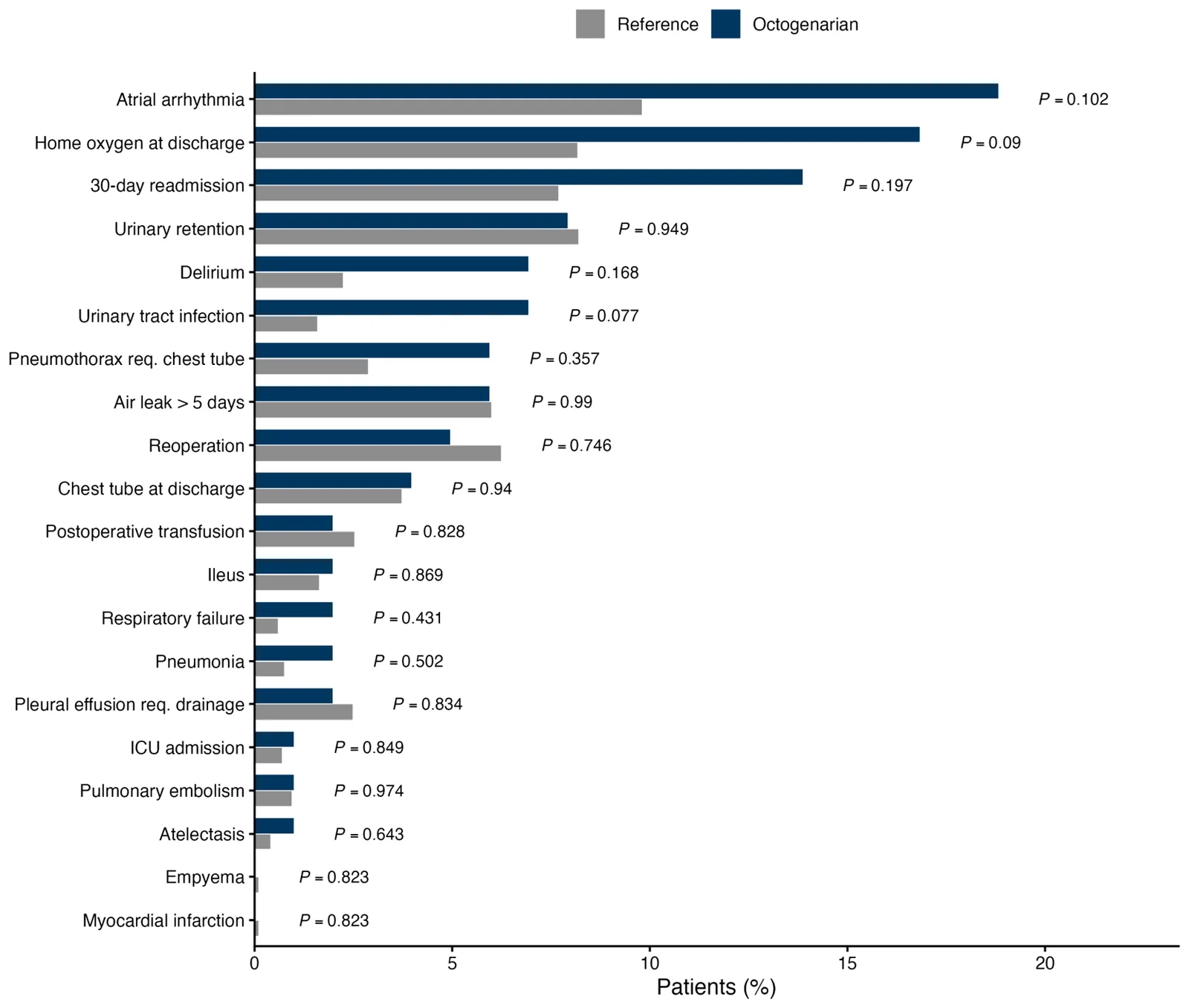
Adjusted comparison of postoperative outcomes.

**Figure 4:**
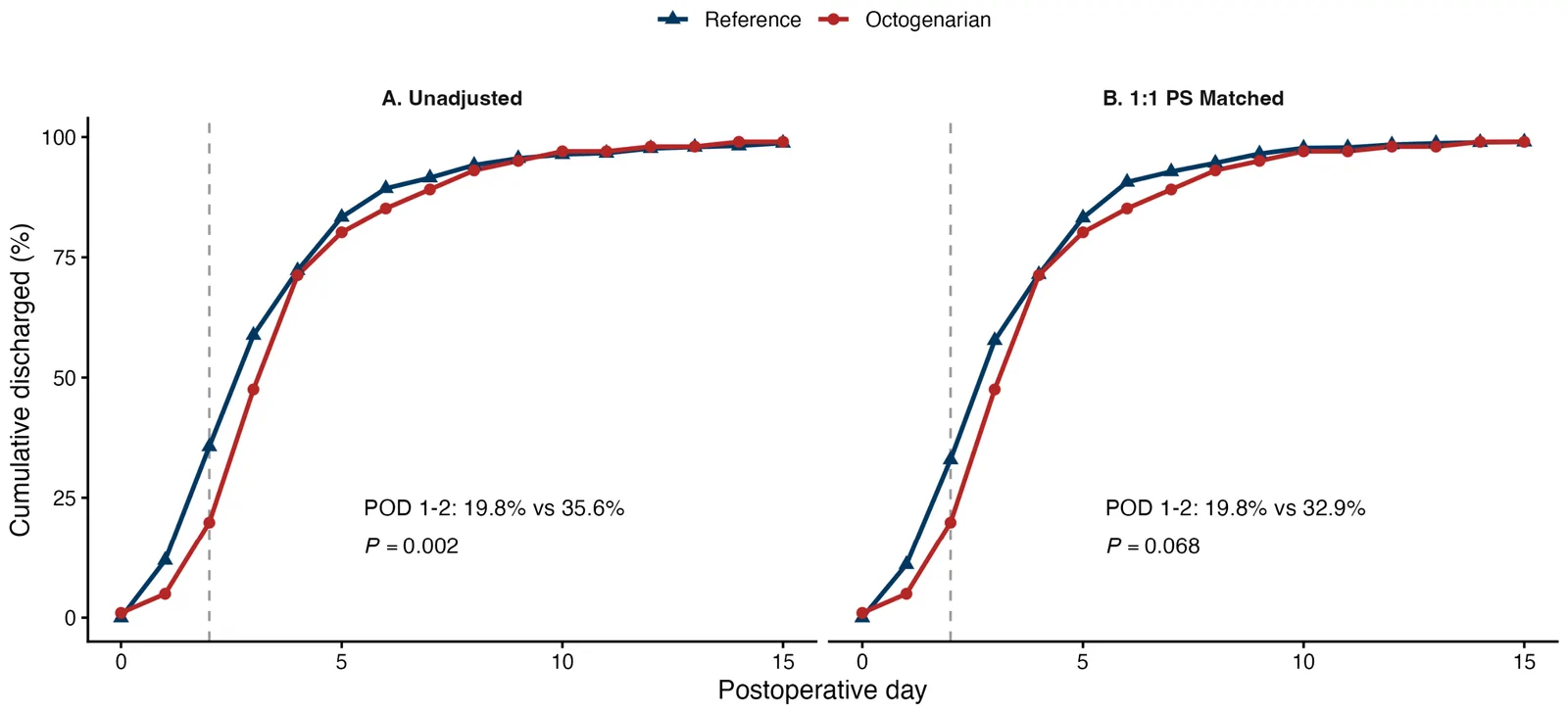
(**A**) Unadjusted time to discharge vs. (**B**) adjusted time to discharge.

**Table 1:** Baseline patient characteristics.

Characteristic	Octogenarian (*n* = 101)	Reference (*n* = 721)	*p*-Value
Age, mean (SD), y	82.4 (2.2)	69.7 (3.1)	<0.001
Male sex	49 (48.5)	342 (47.4)	0.915
Race			0.088
White	97 (96.0)	650 (90.2)	
Black/African American	2 (2.0)	53 (7.4)	
Asian	2 (2.0)	8 (1.1)	
Other	0 (0.0)	10 (1.4)	
BMI, median [IQR], kg/m^2^	27.3 [23.9, 30.6]	27.5 [24.4, 31.1]	0.536
Smoking status			<0.001
Current	8/94 (8.5)	209/669 (31.2)	
Former	67/94 (71.3)	425/669 (63.5)	
Never	19/94 (20.2)	35/669 (5.2)	
Pack-years, median [IQR]	31.0 [16.0, 50.0]	40.0 [25.0, 55.0]	0.013
FEV_1_, median [IQR], % pred	91.0 [81.0, 108.0]	84.0 [72.0, 97.0]	<0.001
DLCO, median [IQR], % pred	82.0 [66.0, 92.0]	79.0 [65.0, 93.0]	0.839
Creatinine, median [IQR], mg/dL	1.0 [0.8, 1.2]	0.9 [0.7, 1.0]	<0.001
Hemoglobin, median [IQR], g/dL	13.2 [11.9, 14.2]	13.5 [12.5, 14.5]	0.020
ASA class			0.072
II	0 (0.0)	29 (4.0)	
III	92 (91.1)	640 (88.8)	
IV	9 (8.9)	52 (7.2)	
ECOG, mean (SD)	1.1 (0.6)	1.0 (0.5)	0.002
Independent functional status	96 (95.0)	705 (97.8)	0.165
Hypertension	60 (59.4)	461 (63.9)	0.380
Coronary artery disease	27 (26.7)	159 (22.1)	0.310
Prior myocardial infarction	12 (11.9)	43 (6.0)	0.033
Congestive heart failure	7 (6.9)	18 (2.5)	0.025
History of atrial fibrillation	10 (9.9)	31 (4.3)	0.025
Pulmonary hypertension	2 (2.0)	5 (0.7)	0.208
Interstitial lung disease	2 (2.0)	5 (0.7)	0.208
Peripheral vascular disease	9 (8.9)	53 (7.4)	0.548
Cerebrovascular history	5 (5.0)	28 (3.9)	0.587
Diabetes mellitus	15 (14.9)	157 (21.8)	0.118
Home oxygen	2 (2.0)	18 (2.5)	>0.999
Segmentectomy (vs lobectomy)	9 (8.9)	91 (12.6)	0.332
Cancer type			0.520
Adenocarcinoma	66 (65.3)	465 (64.5)	
Squamous cell	23 (22.8)	191 (26.5)	
Other	12 (11.9)	65 (9.0)	
Clinical T stage			0.007
T1	53/87 (60.9)	452/597 (75.7)	
T2	26/87 (29.9)	96/597 (16.1)	

Data are mean (SD) or median [IQR] for continuous variables and *n* (%) for categorical variables. Where denominators differ from the column total, data were missing. Continuous variables were compared with the Wilcoxon rank-sum test; categorical variables with Fisher’s exact test (Fisher–Freeman–Halton extension for multicategory variables). ASA, American Society of Anesthesiologists physical status; BMI, body mass index; DLCO, diffusing capacity for carbon monoxide; ECOG, Eastern Cooperative Oncology Group performance status; FEV_1_, forced expiratory volume in 1 s.

**Table 2:** Unadjusted postoperative outcomes.

Outcome	Octogenarian (*n* = 101)	Reference (*n* = 721)	*p*-Value
Air leak > 5 days	6 (5.9)	46 (6.4)	>0.999
Atelectasis	1 (1.0)	5/719 (0.7)	0.547
Pleural effusion	2 (2.0)	13 (1.8)	0.706
Pneumonia	2 (2.0)	9 (1.2)	0.634
Respiratory failure	2 (2.0)	10 (1.4)	0.650
Pulmonary embolism	1 (1.0)	3 (0.4)	0.409
Pneumothorax requiring chest tube	6 (5.9)	16 (2.2)	**0.043**
Atrial arrhythmia	19 (18.8)	66 (9.2)	**0.005**
Myocardial infarction	0 (0.0)	1 (0.1)	>0.999
Ileus	2 (2.0)	10 (1.4)	0.650
Urinary tract infection	7 (6.9)	12/718 (1.7)	**0.005**
Urinary retention	8 (7.9)	43/718 (6.0)	0.507
Empyema	0 (0.0)	2 (0.3)	>0.999
Delirium	7 (6.9)	22/718 (3.1)	0.076
Postoperative transfusion	2 (2.0)	15 (2.1)	>0.999
Major complication	9 (8.9)	49 (6.8)	0.409
Reoperation	5 (5.0)	32 (4.4)	0.797
ICU admission	1 (1.0)	14 (1.9)	>0.999
Postoperative LOS, days	4.0 [3.0, 5.0]	3.0 [2.0, 5.0]	**0.012**
Chest tube at discharge	4 (4.0)	31/720 (4.3)	>0.999
Home oxygen at discharge	17 (16.8)	85 (11.8)	0.149
30-day readmission	14 (13.9)	58/720 (8.1)	0.061
30-day mortality	0 (0.0)	2/720 (0.3)	>0.999
Rapid discharge (POD ≤ 2)	20 (19.8)	257 (35.6)	**0.002**

Data are median [IQR] for LOS and *n* (%) for all other outcomes. Where denominators differ from the column total, data were missing. Continuous outcomes were compared with the Wilcoxon rank-sum test, and categorical outcomes with Fisher’s exact test. A two-sided *p* < 0.05 was considered significant. ICU, intensive care unit; LOS, length of stay; POD, postoperative day.

**Table 3:** Propensity score 1:1 matched comparison of postoperative outcomes.

Outcome	Octogenarian (%)	Reference (%)	Difference (95% CI)	*p*-Value
Air leak > 5 days	5.9	6.0	0.0 (−7.5 to +7.4)	0.990
Atelectasis	1.0	0.4	+0.6 (−1.9 to +3.1)	0.643
Pleural effusion	2.0	2.5	−0.5 (−5.1 to +4.1)	0.834
Pneumonia	2.0	0.7	+1.2 (−2.4 to +4.9)	0.502
Respiratory failure	2.0	0.6	+1.4 (−2.1 to +4.8)	0.431
Pulmonary embolism	1.0	0.9	0.0 (−2.9 to +3.0)	0.974
Pneumothorax requiring chest tube	5.9	2.9	+3.1 (−3.5 to +9.6)	0.357
Atrial arrhythmia	18.8	9.8	+9.0 (−1.8 to +19.8)	0.102
Myocardial infarction	0.0	0.1	−0.1 (−1.0 to +0.8)	0.823
Ileus	2.0	1.6	+0.3 (−3.8 to +4.5)	0.869
Urinary tract infection	6.9	1.6	+5.3 (−0.6 to +11.3)	0.077
Urinary retention	7.9	8.2	−0.3 (−8.3 to +7.8)	0.949
Empyema	0.0	0.1	−0.1 (−1.0 to +0.8)	0.823
Delirium	6.9	2.2	+4.7 (−2.0 to +11.4)	0.168
Postoperative transfusion	2.0	2.5	−0.5 (−5.4 to +4.4)	0.828
Major complication	8.9	8.1	+0.8 (−8.8 to +10.4)	0.871
Reoperation	5.0	6.2	−1.3 (−9.1 to +6.5)	0.746
ICU admission	1.0	0.7	+0.3 (−2.8 to +3.3)	0.849
Postoperative LOS, days	4.0 [3.0, 5.0]	3.0 [2.0, 5.0]	+0.4 (−0.5 to +1.3) days	0.369
Chest tube at discharge	4.0	3.7	+0.2 (−6.1 to +6.6)	0.940
Home oxygen at discharge	16.8	8.2	+8.7 (−1.4 to +18.7)	0.090
30-day readmission	13.9	7.7	+6.2 (−3.2 to +15.6)	0.197
30-day mortality	0.0	0.1	−0.1 (−1.2 to +0.9)	0.781
Rapid discharge (POD ≤ 2)	19.8	32.9	−13.1 (−27.2 to +1.0)	0.068

Values are pooled estimates averaged across 20 matched imputed datasets, each containing 101 patients per group; LOS is the median [IQR]. Differences are octogenarian minus reference or absolute risk differences in percentage points, or mean differences in days for LOS are estimated by linear models on the matched sample, with 95% confidence intervals and *p*-values pooled by Rubin’s rules and derived from the same model. A two-sided *p* < 0.05 was considered significant. CI, confidence interval; ICU, intensive care unit; LOS, length of stay; POD, postoperative day.

## Data Availability

The raw data supporting the conclusions of this article may be made available by the authors upon request.

## Supplementary Materials

The following supporting information can be downloaded at https://www.mdpi.com/article/10.3390/cancers18193089/s1. Figure S1: Covariate balance using multiple imputations; Table S1: Distribution of operations by calendar period; Table S2: Missing data; Table S3: Covariate balance before and after 1:1 propensity-score matching.
