# Supplementary material for: Rethinking Age Limits for Robotic Lung Cancer Resection: Contemporary Short-Term Outcomes in Octogenarians
Source: Cancers (Basel). 2026 Sep 23;18(19):3089. doi: 10.3390/cancers18193089 (PMC13663933; doi:10.3390/cancers18193089)
Supplement: Supplementary file 1 [file cancers-18-03089-s001.zip › cancers-4495066-supplementary.pdf]

Supplemental Figure S1. Covariate balance using multiple imputations.

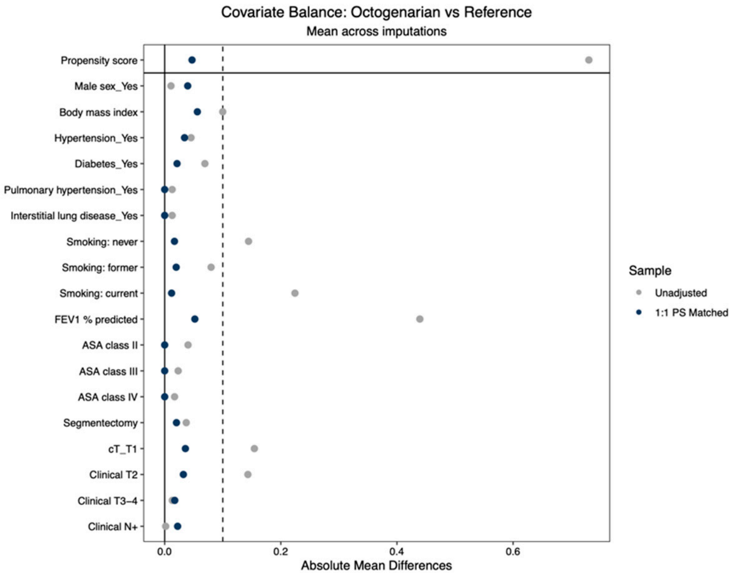

Supplemental Table S1. Distribution of operations by calendar period.

Year	Octogenarian	Reference	Total	% Octogenarian
2015	2	24	26	7.7
2016	1	30	31	3.2
2017	8	48	56	14.3
2018	8	52	60	13.3
2019	16	77	93	17.2
2020	15	57	72	20.8
2021	13	62	75	17.3
2022	7	80	87	8.0
2023	10	97	107	9.3
2024	13	85	98	13.3
2025	8	88	96	8.3
2026	0	21	21	0.0
**2015–2018**	**19**	**154**	**173**	**11.0**
**2019–2022**	**51**	**276**	**327**	**15.6**
**2023–2026**	**31**	**291**	**322**	**9.6**

Median year of surgery was 2021 for octogenarians and 2022 for the reference cohort (P=0.12); the distribution across eras did not differ significantly (P=0.058).

Supplemental Table S2. Missing data.

Variable	Octogenarian (n=101)	Reference (n=721)	Overall (n=822)
Clinical T stage	14 (13.9)	124 (17.2)	138 (16.8)
Pack-years	29 (28.7)	120 (16.6)	149 (18.1)
DLCO, % predicted	10 (9.9)	89 (12.3)	99 (12.0)
Smoking status	7 (6.9)	52 (7.2)	59 (7.2)
Creatinine	3 (3.0)	31 (4.3)	34 (4.1)
Hemoglobin	2 (2.0)	31 (4.3)	33 (4.0)
FEV_1, % predicted	2 (2.0)	10 (1.4)	12 (1.5)
Clinical N stage	0 (0.0)	1 (0.1)	1 (0.1)
Urinary tract infection	0 (0.0)	3 (0.4)	3 (0.4)
Urinary retention	0 (0.0)	3 (0.4)	3 (0.4)
Delirium	0 (0.0)	3 (0.4)	3 (0.4)
Atelectasis	0 (0.0)	2 (0.3)	2 (0.2)
Chest tube at discharge	0 (0.0)	1 (0.1)	1 (0.1)
30-day readmission	0 (0.0)	1 (0.1)	1 (0.1)
30-day mortality	0 (0.0)	1 (0.1)	1 (0.1)

Data are n (%). All other baseline covariates and postoperative outcomes were complete. Clinical staging was not recorded institutionally before 2019.

Supplemental Table S3. Covariate balance before and after 1:1 propensity-score matching.

Covariate	Type	SMD before	SMD after
Propensity	Distance	0.730	0.047
Male sex	Binary	0.011	0.029
Body mass index	Continuous	−0.100	0.002
Hypertension	Binary	−0.045	0.007
Diabetes mellitus	Binary	−0.069	−0.010
Pulmonary hypertension	Binary	0.013	0.000
Interstitial lung disease	Binary	0.013	0.000
Smoking status: never	Binary	0.144	−0.003
Smoking status: former	Binary	0.080	0.002
Smoking status: current	Binary	−0.224	0.001
FEV_1, % predicted	Continuous	0.439	0.014
ASA class II	Binary	−0.040	0.000
ASA class III	Binary	0.023	0.000
ASA class IV	Binary	0.017	0.000
Segmentectomy	Binary	−0.037	0.002
Clinical T1	Binary	−0.154	−0.031
Clinical T2	Binary	0.143	0.025

Clinical T3–T4	Binary	0.011	0.005
Clinical node-positive	Binary	0.002	0.006

Standardized mean differences (SMDs) averaged across the 20 imputed datasets. Absolute SMD <0.10 was considered adequate balance. All 19 covariates achieved balance.
